# Artificial Intelligence for Upper Gastrointestinal Endoscopy: A Roadmap from Technology Development to Clinical Practice

**DOI:** 10.3390/diagnostics12051278

**Published:** 2022-05-21

**Authors:** Francesco Renna, Miguel Martins, Alexandre Neto, António Cunha, Diogo Libânio, Mário Dinis-Ribeiro, Miguel Coimbra

**Affiliations:** 1 Instituto de Engenharia de Sistemas e Computadores, Tecnologia e Ciência, 3200-465 Porto, Portugal; miguel.l.martins@inesctec.pt (M.M.); alexandre.h.neto@inesctec.pt (A.N.); acunha@utad.pt (A.C.); mcoimbra@fc.up.pt (M.C.); 2 Faculdade de Ciências, Universidade do Porto, 4169-007 Porto, Portugal; 3 Escola de Ciências e Tecnologia, Universidade de Trás-os-Montes e Alto Douro, Quinta de Prados, 5001-801 Vila Real, Portugal; 4 Departamento de Ciências da Informação e da Decisão em Saúde/Centro de Investigação em Tecnologias e Serviços de Saúde (CIDES/CINTESIS), Faculdade de Medicina, Universidade do Porto, 4200-319 Porto, Portugal; diogo.monteiro@ipoporto.min-saude.pt (D.L.); mario.ribeiro@ipoporto.min-saude.pt (M.D.-R.)

**Keywords:** artificial intelligence, deep learning, upper GI endoscopy (UGIE), computer vision, convolutional neural networks

## Abstract

Stomach cancer is the third deadliest type of cancer in the world (0.86 million deaths in 2017). In 2035, a 20% increase will be observed both in incidence and mortality due to demographic effects if no interventions are foreseen. Upper GI endoscopy (UGIE) plays a paramount role in early diagnosis and, therefore, improved survival rates. On the other hand, human and technical factors can contribute to misdiagnosis while performing UGIE. In this scenario, artificial intelligence (AI) has recently shown its potential in compensating for the pitfalls of UGIE, by leveraging deep learning architectures able to efficiently recognize endoscopic patterns from UGIE video data. This work presents a review of the current state-of-the-art algorithms in the application of AI to gastroscopy. It focuses specifically on the threefold tasks of assuring exam completeness (i.e., detecting the presence of blind spots) and assisting in the detection and characterization of clinical findings, both gastric precancerous conditions and neoplastic lesion changes. Early and promising results have already been obtained using well-known deep learning architectures for computer vision, but many algorithmic challenges remain in achieving the vision of AI-assisted UGIE. Future challenges in the roadmap for the effective integration of AI tools within the UGIE clinical practice are discussed, namely the adoption of more robust deep learning architectures and methods able to embed domain knowledge into image/video classifiers as well as the availability of large, annotated datasets.

## 1. Introduction

The Global Burden of Disease [1] estimates that approximately 10 million people died prematurely as a result of cancer in 2017, representing one-sixth of all human deaths in that year. Stomach cancer is particularly lethal, corresponding to the third deadliest type of cancer in the world (0.86 million deaths in 2017), and according to IARC [2], in 2035, a 20% increase will be observed both in incidence and mortality if no countermeasures are adopted. The key to better gastric cancer management is early detection via effective screening strategies that can improve patient survival rates. Minimal invasive screening and endoscopy play a paramount role in early diagnosis and therefore improved survival rates [3]. Esophagogastroduodenoscopy (EGD), also known as upper gastrointestinal (UGI) endoscopy (UGIE), is the gold standard for the diagnosis of diseases of the UGI tract. However, due to cognitive and technical factors, the risk for misdiagnosis is significant [4], with up to 9% of missed lesions. In this scenario, artificial intelligence (AI), and more specifically machine learning, has recently shown great potential in assisting endoscopists in performing UGIE exams and interpreting the corresponding findings. At the core of this “revolution” lies the adoption of modern computer vision algorithms based on the deep learning paradigm. These algorithms have been recently adopted to assist endoscopists in three fundamental tasks: (i) quality assessment, (ii) detection of lesions, and (iii) their characterization.

Quality assessment of endoscopic procedures is a fundamental component of health care nowadays to avoid missing lesions and prevent death. By assuring that the entire stomach was observed, endoscopists must complete the observation of all parts or landmarks [5]. Hereby, AI algorithms can be designed to automatically recognize key anatomical landmarks of the UGI, thus informing the operator regarding the completeness of the examination. Secondly, once a complete inspection of the UGI mucosa is guaranteed, the next question that arises during the examination is if there are any lesions and what is the histological type of the lesion. Additionally, in this case, computer vision algorithms can play a fundamental role in providing an answer to these questions, by indicating the presence of a lesion in a frame or by providing a spatial indication of the portion of the frame that is affected by lesions (object detection). In fact, when assessing missed lesions (false negative) in AI studies, up to 18% were missed [6]. Finally, after successful detection of a lesion, its characterization is fundamental for further patient management. Artificial intelligence can assist the endoscopist with an ancillary aim for visual endoscopy assessment which is an optical biopsy.

There are multiple reviews and meta-analyses on the application of AI for UGIE computer-assisted decision (CAD) in the literature (see, e.g., [7,8,9,10,11,12,13]), often focusing explicitly on clinical needs and on the impact of the current solutions on the clinical practices. In contrast, our work will provide an in-depth analysis of the algorithmic details and experimental methodologies adopted in AI for gastrointestinal endoscopy. It will attempt to establish a pathway to better understand the full potential of AI-based systems tailored to the needs of day-to-day UGIE clinical practice described above: (i) ensuring and evaluating exam completeness via the automatic recognition of standardized UGIE anatomical landmarks (Section 3.1); (ii) automatic detection of gastric lesions (Section 3.2); (iii) automatic characterization of gastric lesions (Section 3.3). A discussion on the obtained results is held in Section 4. Finally, conclusions are drawn in Section 5.

## 2. Methods

Articles included in this review were searched using a variety of queries in the following databases: Pubmed, Scopus, Google Scholar. Articles had to be written in English and have a clear methodology outlining the design of experiments and validations of the proposed models. Data used for the training of the models had to be collected from UGIE in a real clinical setting. Results from clinical trials were only included for illustrative purposes when they measured the performance of humans with and without AI. Since this paper emphasizes the algorithmic procedures, we do not elaborate on the outcomes of these trials. Concerning UGIE exam quality assessment (Section 3.1), we enforced a minimum of 4 anatomical landmark classes for the UGI. As such, we excluded the body of work pertaining to the 2017 MediaEval’s Multimedia for Medicine task (3 anatomical landmarks) [14,15]. Regarding stomach lesion detection in UGIE (Section 3.2), we only consider deep learning-based solutions for gastric lesions. Thus, we discarded [16,17,18,19,20] since they focused on esophageal lesions. Finally, with regard to lesion characterization in the UGIE (Section 3.3), we only included articles published from 2018 onwards that included gastric lesion characterization. Consequently, we excluded [21,22,23] from this review since their analysis was only relevant for anatomical regions different from the stomach.

## 3. Findings

In this section, we will provide an exposition of our findings given the above-mentioned criteria (Section 2). We start by providing a brief motivation for each problem, followed by (i) a critical elaboration on the algorithmic solutions reviewed. We then follow up with (ii) an analysis of the datasets collected to support the AI solutions (number of frames, modalities, number of classes, etc.) and close by (iii) reporting the results as measured by the authors. The outcomes of this review are summarized in Table 1, Table 2, Table 3 and Table 4.

### 3.1. UGIE Exam Quality Assessment

Human factors can impair the effectiveness of UGIE in the early detection of gastric lesions [4]. In particular, poor patient tolerance, quick inspection, operator distraction, and anatomically challenging areas (blind spots) can contribute to the failure of observing the entire stomach. Thus, avoiding blind spots is a fundamental prerequisite for the efficacy of UGIE in detecting early-stage gastric cancer [24,25].

**Figure 1:**
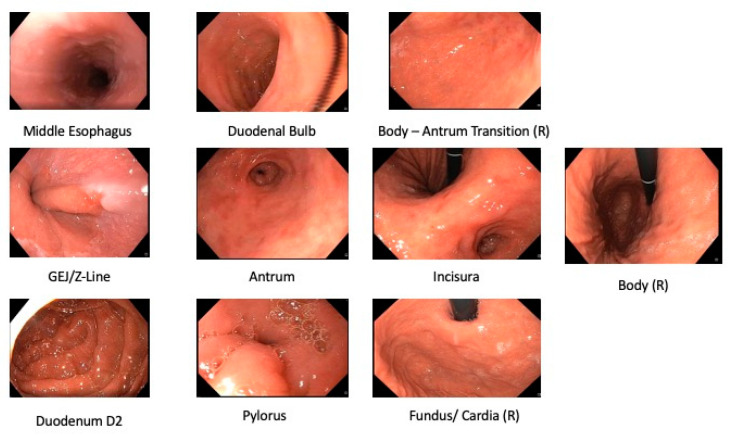
Examples of key anatomical landmarks, containing (from **left** to **right**, **top** to **bottom**): the middle esophagus, gastroesophageal (GEJ) junction and Z-Line, second portion (D2) of duodenum, antrum, pylorus, the transition from the antrum in the gastric body, the incisura, fundus and cardia in retroflexed view (R) and the body in retroflex view (R).

Photodocumentation can guarantee complete examination. Photodocumentation and quality guidelines have been issued. Increasing the photodocumentation completion rate was set forth as one of the top priorities [26,27] of both the European society of gastrointestinal endoscopy (ESGE) [28,29] and the American Society of Gastrointestinal Endoscopy (ASGE) [30]. The British Society of Gastroenterology (BSG) and the ESGE recommend the inclusion of at least 10 photos from 8 anatomical locations in the UGIE report [31,32] (See Figure 1). Since the lesion detection rate has been shown to correlate with the number of photos taken [33], the Japanese Society of Gastroenterological Cancer Screening (JSGCS) recommends a more complete protocol [34]. However, due to its complexity, this protocol was put into practice only in Japan [24]. In 2013, Kenshi Yao [24] proposed a simplification of the Japanese protocol in the form of the Systematic Screening Protocol for Stomach (SSS), with the objective of leveraging adequate complexity with sufficiently high-quality mapping of the stomach (22 anatomical sites) [24]. An extended version of the protocol with two additional landmarks was shown to be also effective for screening lesions in the stomach [35]. In 2020, the World Endoscopy Organization (WEO) issued a position statement [36] where a new standard for UGIE examination was proposed, the systematic alphanumeric coded endoscopy (SACE). This standard expanded on the SSS protocol by requiring that endoscopists document 28 unique anatomical locations, including key anatomical sites from the hypopharynx to the second portion of the duodenum. Examples of normal anatomical landmarks are reported in Figure 1.

AI solutions based on anatomical landmark detection have been developed to assist in the generation of UGIE photodocumentation and evaluation of exam completeness. This task has recently gathered the interest of the computer vision and machine learning communities, as testified by the organization of events such as the MediaEval’s 2017 Multimedia for Medicine task [37], where 3 anatomical classes gathered from real UGIE data had to be correctly classified, together with diseased tissue, from the Kvasir dataset [38]. Several solutions for automatic anatomical landmark detection have been proposed, the majority of which are based on the application of different deep convolutional neural network (CNN) architectures.

**Figure 2:**
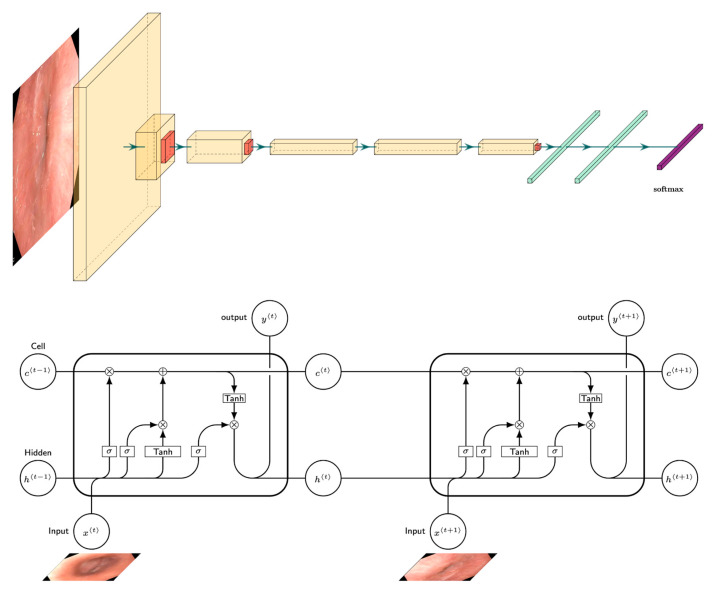
(**Top**) Convolutional single-frame algorithm versus (**Bottom**) recurrent multi-frame algorithm. In single-frame algorithms, frames sampled from UGIE images are processed independently. In multi-frame approaches, the frames sampled from UGIE videos are processed in sequence.

We group the UGIE landmark detection methodologies into two groups: single and multi-frame algorithms (Figure 2). In the former (Section 3.1.1), the task of anatomical site detection is treated as a simple multi-class image classification problem. The latter (Section 3.1.2) addresses computer-assisted tools for blind spot mitigation, where multiple frames are considered, thus leveraging the sequential nature of the UGIE videos in the classification task.

#### 3.1.1. Anatomical Landmark Detection UGIE—Single Frame

A first series of approaches that appeared in the literature for anatomical landmark detection in UGIE were based on the application of deep learning techniques that classified each frame of an UGIE video independently from the others. Such approaches were based on the adaptation and fine-tuning of existing image classification models in order to identify anatomical landmarks.

A summary of the works that have proposed single-frame anatomical landmark detection approaches can be found in Table 1.

**Table 1:** Single-frame algorithms for anatomical landmark detection.

Authors	Data Annotation Protocol	Dataset	Classes	Algorithm	Pre-Processing	Validation	Average Performance	AI Impact (Clinical Setting)
*Takiyama* et al., *2018* [39]	Undefined	(*Private Dataset*) 44,416 UGIE images (optimal view WLI)	4 sites + 3 gastric sites	GoogleLeNet	Black frame cropping	Holdout set	(4 anatomical classes) Accuracy: 99% Sensitivity: 94% (from 87% to 99%) 96% (from 96% to 97%) (3 gastric sub-classes) Sensitivity: 97% (from 96% to 97%) Specificity: 98% (from 98% to 99%)	*N/A*
*Wu* et al., *2019* [40]	2 experts with >10 years of experience	(*Private Dataset*) 24,549 WLI images	10 or 26 sites	VGG16-Resnet50	CNN filters blurry frames	Holdout set	Accuracy: 90% (10 sites) 66% (26 sites)	(Single-center, retrospective trial) [40] **** Endoscopist accuracy: 90% (10 sites, experts) 63% (26 sites, experts) 87% (10 sites, seniors) 59% (26 sites, seniors) 83% (10 sites, novices) 46% (26 sites, novices)
*Zhang Xu* et al., *2019* [41]	2 expert endoscopists (years of experience unknown)	(*Private Dataset*) 75,275 UGIE images (including non-informative and NBI frames) *	10 sites + uninformative + NBI	Muli-Task Custom CNN + SSD	None	Holdout set	Average precision (mAP): 94%	*N/A*
*He* et al., 2019 ** [42]	1 doctoral student 1 clinical gastroenterology research fellow	3704 UGIE images (WLI+LCI frames) optimal views	11 sites + N/A	Inception-v3	Data-driven ROI cropping	5-fold C.V.	Accuracy: 83% F1: 80% (from 53% to 94%)	*N/A*
*Igarashi* et al., *2020* [43]	1 expert with >30 years of experience 1 endoscopists with >4 years of experience	(*Private Dataset*) 85,246 upper GI images	10 sites from UGIE + 4 classes pertaining to specimens and other examinations	AlexNet	None	Holdout set	Accuracy: 97%	*N/A*
*Sun* et al., 2021 *** [44]	>1 endoscopist with >5 years of experience	(*Private Dataset*) 10,474 UGIE images including NBI	11 sites + NBI	Custom CNN+RCF	ROI extraction + bilinear interpolation	5-fold C.V.	Accuracy: 99% Precision: 93% F1 score: 92%	*N/A*
*Chang* et al., *2021* [27]	Unclear	(*Private Dataset*) 15,723 frames from asymptomatic patients	8 classes	ResNeSt	None	Holdout set	Accuracy: 97%	*N/A*

* Addressed in different classification tasks. ** We only consider the protocol with all the landmarks and N/A classes in this article. *** We considered only the values for the first ResNeSt, since the ampulla was divided into two categories and trained with a second model and different dataset. **** Only findings concerning blind spots were considered.

##### Algorithms

Standard, well-known deep convolutional neural networks were used by five of the works considered in this review, including the seminal AlexNet architecture [43,44], VGG architectures [40,42], Inception architectures [39,42,44], and ResNet [40,42,44]. Two works considered more recent CNN models, including the DenseNet [42] and a split-attention network (ResNeSt) [27]. DenseNet approaches aim to improve the generalization of the model and reduce the problem of vanishing gradients via the use of multiple, dense connections between each layer and the following layers in the network. On the other hand, the ResNeSt [41] applied channel-wise attention mechanisms on different network branches to leverage their success in capturing cross-feature interactions and learning diverse representations. The authors of [44] also considered a more sophisticated approach that combined standard CNN architectures with richer convolution features (RCF) [45]. In particular, the RCF hierarchy was used to extract feature maps from each convolution using 12 convolutional filters with 1 × 1 kernels. The feature maps from each stage of the CNN were then aggregated by summation, thus establishing a one-to-one correspondence between the channel and the landmark site. Of particular relevance and in contrast with all the remaining approaches considered within this section, [41] was not limited to the task of frame classification. It considered an object detection problem, thus providing as output also a bounding box defining the region of the image associated with the landmark. This was achieved by using a single-shot multibox detector (SSD) model.

**Figure 3:**
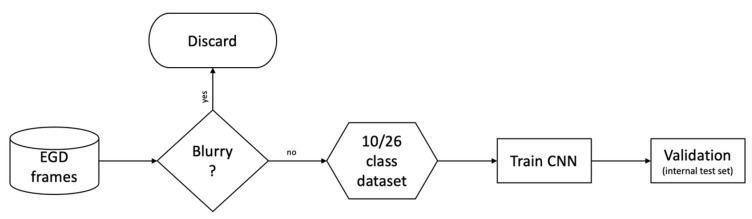
The single-frame classifier pipeline proposed by Wu et al. [40]. Note that there are two separate classification objectives with respect to anatomical locations: one considering 10 anatomical sites and another considering 26 anatomical sites (adapted from [40]).

In three cases, frames extracted from UGIE were classified directly using deep learning models with almost no preprocessing steps. Exceptions to this are represented by [44], which identifies the region of interest (ROI) of each frame before classification using image clipping, and [39], which cropped the black frame surrounding each image. Instead of applying standard preprocessing, two of the considered works included a “filtering” step where a CNN is trained to first select the frames of interest from the video data stream. This step aims at discriminating between informative and non-informative images [41] or filtering out blurry images [40] (see Figure 3).

Data augmentation has also been explicitly considered in two works by applying random transformations to the training images. Such transformations included random brightness and contrast enhancement [44], random rotations, and zooms [39].

Few efforts have been made so far to provide explainable and interpretable models for anatomical landmark detection. Namely, only class activation maps (CAM) methods have been applied in two cases [40,44] to highlight which parts of the image were mostly responsible for the output class provided by the model.

From the analysis of the algorithmic approaches currently used for single-frame anatomical landmark detection, it is possible to conclude that standard CNN architectures have the potential to correctly recognize anatomical landmarks in UGIE video frames. On the other hand, the existing solutions are mainly focused on the adoption of CNN models that have been proposed to the computer vision community almost a decade ago. In this sense, further explorations could be directed to a more systematic use of data augmentation approaches and towards the design of more interpretable models, able to provide more reliable outputs to the endoscopist. Finally, the current solutions seem not to push for the integration of domain knowledge into the adopted data-driven models.

##### Datasets

All the considered works used private data to train and validate the models. The datasets varied considerably in terms of size, the types of imaging modalities, and the number of classes associated with different anatomical landmarks (see Table 1). Dataset size ranged from a few thousand (e.g., 2459 in [40]) to tens of thousands (e.g., 85,246 in [43]).

The total number of classes (anatomical landmarks) changed from a reduced number in preliminary studies to complete protocols. Namely, [39] focused on the classification of only the four main organs in the upper GI tract, i.e., larynx, esophagus, stomach, and duodenum. It also tested a further splitting of the class of stomach into three further regions: upper, middle, and lower stomach. Other custom protocols were adopted, some with 10 anatomical locations [41], 11 anatomical locations [42], 10 and 26 landmarks [40], 12 landmarks [44], and 14 locations [43]. The official photodocumentation protocol put forward by the ESGE was considered in [27]. Four works also introduced explicitly a class named N/A, which contained frames that were considered noisy or not informative [27,42,43], or that included different imaging modalities [41], or pathological findings that could alter the appearance of anatomical structures [42].

Three works considered only non-magnified white light imaging (WLI) frames [39,40,41]. One work considered WLI, narrow band imaging (NBI) and Blue Laser Imaging (BLI) data [44]. Linked colored imaging (LCI) data was considered in [34]. Furthermore, one work included in the analysis images obtained with local coloring techniques, such as indigo carmine (IC) or iodine [43].

The analysis of the datasets used to train and validate models for single-frame anatomical landmark detection suggests a large variability in the definition of these studies. This fact prevents to perform a fair comparison between the different approaches, thus limiting the validity of conclusions derived from such comparisons. First, it is important to note that all datasets are private, leading to limited reproducibility of the proposed results and raising potential concerns in terms of the introduction of bias in the data (e.g., selection bias, spectrum bias, overfitting bias [10]). In addition, the definition of anatomical landmark classes is largely non-uniform, which implies that the classification problems addressed by the different proposed models can have different degrees of complexity. This again prevents to draw strong conclusions from a comparative analysis.

##### Results

In general, it is not possible to draw statistically significant conclusions regarding the comparison of the performance obtained by the different methods considered for single-frame anatomical landmark detection due to the significant differences in the datasets used for training and validations. These differences involve the number of images used, the number of anatomical landmarks considered, restrictions in the imaging modalities adopted, and pre-selection of informative frames. Thus, the performance of the different classification tasks cannot be directly compared.

On the other hand, as supported by Table 1, the considered works report an average classification performance with sensitivity and specificity values often in excess of 90%, which is a positive indicator of the relevance of AI algorithms for anatomical landmark detection. Nevertheless, some limitations can be registered when classifying some of the anatomical landmarks due to their resemblance and shared features. For example, [39] observed classification errors in the discrimination of images belonging to the larynx and esophagus, even if the morphological structure observed is sufficient to be distinguished by the human eye. Other errors involved misclassification of contiguous landmarks (e.g., esophagus and upper stomach, lower stomach, and duodenum [39]). Furthermore, images not contained in the N/A class were often wrongly assigned to this class, due to its high intra-class variability [43].

It is also worth mentioning that, in three cases, video frames from UGIE videos were preventively excluded, for example, when they focused on pathological findings [39], contained food residue or bleeding [39,42], when they were not considered in focus or were affected by blurring effects [39]. In this sense, the results showcased by such works might not reflect directly the performance that is possible to achieve in real-world clinical scenarios.

#### 3.1.2. Anatomical Landmark Detection in UGIE—Multiple Frames

Although deep learning classifiers have shown very promising results when detecting anatomical sites in the UGI, they do not account for temporal correlations among the frames. In fact, the problem of segmenting frames in the temporal dimension to a set of classes provides a closer description of the task of mapping the UGI tract to a set of key anatomical locations given an UGIE video. Algorithms that take this modality into consideration have become the new standard for anatomical landmark detection in UGIE videos.

A summary of the works that have proposed multi-frame anatomical landmark detection approaches can be found in Table 2.

**Table 2:** Multi-frame algorithms for anatomical landmark detection.

Authors	Data Annotation Protocol	Dataset	Classes	Algorithm	Augmentation	Validation	Average Performance	AI Impact (Clinical Setting)
*Wu* et al., *2019* [45]	2 seniors 1–5 years of experience 3 experts > 5 years of experience 2 doctoral students **	*(Private Dataset)* 34,513 UGIE images 107 UGIE videos	26 sites + N/A	VGG-16 + DRL	None	10-fold C.V.	Accuracy: 90% (from 70% to 100%) Sensitivity: 88% (from 63% to 100%) Specificity 95% (from 75% to 100%)	(Single-center randomized controlled trial) [45] *** Blind spot rate (#Landmarks) Humans+Algorithm: 5.86 ± 6.89 Humans (control): 22.46 ± 14.38 (Mean performance improvement: 15.39 from 19.23 to 11.54) (Multicenter Randomized Controlled Trial) Wu et al., 2021 [46] Blind spot rate (#Landmarks): Humans+Algorithm: 5.38 ± 4.32 Humans (control): 9.82 ± 9.82 (Prospective, single-blind, 3-parallel-group, randomized, single-center trial) Chen et al., 2019 [47] *** Blind spot rate (#Landmarks): Humans+Algorithm: 3.42 ± 5.73 Humans (control): 22.46 ± 14.76
*Choi* et al., *2020* [26]	5 blinded seniors with >3 years of experience	*(Private Dataset)* 2599 images from 250 UGIEs	8 sites	SENet + positional loss	Random scaling, cropping, rotation and horizontal flip	10-fold C.V.	Accuracy: 98% (from 94% to 99%) Sensitivity: 97% (from 94% to 99%) Specificity: ~100%	*N/A*
*Ding* et al., *2021* [48]	UGIE images from clinical reports	*(Private Dataset)* 3605 UGIE anatomical site frames 2346 background class frames	6 sites + background	EfficientNet-b3 + thresholded sliding window with exponential decay	Random shear, scaling and translation	Holdout set and weighted oversampling	Accuracy: 88% * Precision: 89% * (from 65% to 95%) Recall: 88% * (from 69% to 99%)	*N/A*
*Yan-Dong Li* et al., *2021* [47]	3 blinded seniors with 5–10 years of experience 1 expert > 10 years of experience	170,297 UGIE *(Private Dataset)* images 5779 UGIE videos	31 sites	Inception-v3 + LSTM	Random HSV jittering and corner cropping	Holdout set	Accuracy: 95% (from 88% to 100%) Sensitivity: 96% (from 81% to 100%) Specificity: 99% (from 99% to 100%)	*N/A*

* Measured on isolated still frames that do not necessarily belong to the same UGIE video. Background class included. ** Doctoral students only classified unqualified frames in the N/A class for the anatomical site classification task. *** Only findings regarding blind spots in conventional EGD were included; human level of expertise unknown.

##### Algorithms

Two different main classes of algorithmic approaches were observed when including temporal correlation in anatomical landmark detection approaches: (i) sequential classification [26,48] and (ii) temporal deep learning models [45,49]. In particular, [26] focused on the task of predicting if the UGIE was complete or not. This problem has been tackled by first training a CNN, more specifically, a squeeze-and-excitation network (SENet), to classify single frames into the corresponding anatomical landmark classes. Then, the output sequence of prediction probabilities was processed in order to determine the completeness of the procedure. A slightly more sophisticated approach for the combination of CNN outputs was implemented in [48]. Additionally, in this case, the first step in this method consists of the training of a CNN model for single-frame classification. In particular, the EfficientNet [50] model was adopted. Then, a sliding window with exponential decay was used to weigh the prediction probabilities of 5 neighboring frames, thus providing a smoothing effect able to mitigate the impact of noisy frames.

**Figure 4:**
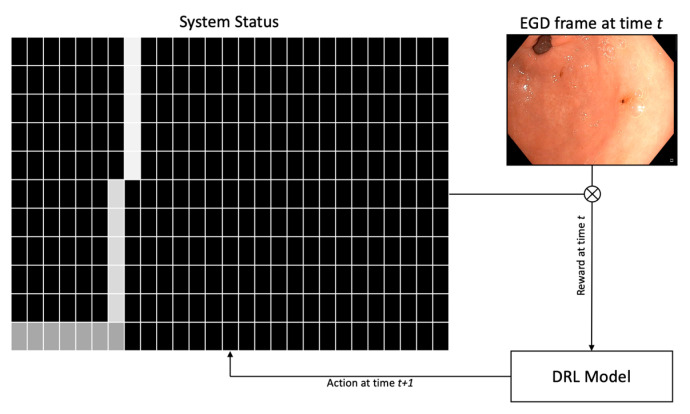
The DRL scheme adopted in [45]. There are 27 possible states concerning anatomical locations and N/A. The last row shows the observed classes until time *t*. At each timestep, a new prediction is displayed on the top row, with the color-coded confidence score. The lighter the shade, the higher the confidence (adapted from [45]).

More sophisticated temporal deep learning models also include a first step for training single-frame CNNs classification. On the other hand, their outputs are then combined using more complex statistical approaches that integrate other neural networks architectures. In particular, the seminal work [45] proposed a system that implements deep reinforcement learning (DRL), which was dubbed WISENSE. A VGG-16 model was trained to classify frames independently. Its predictions were used to compute the landmark class probability for each subsequent frame (see Figure 4). Such predictions were used to generate a 10 × 26 matrix that represented the current state of the DRL system by including information about the probability of the last ten frames being associated with each of the 26 anatomical landmarks considered. The DRL algorithm then take some action based on the current state, thus lighting one of the 26 sites or remaining silent. During the training phase, correct actions are rewarded while incorrect ones are penalized, thus encouraging the system to classify correctly frames containing anatomical landmarks on the basis of the last 10 frames visited. Finally, [48] combines the single-frame classification outputs provided by an Inception-v3 CNN using a long short-term memory (LSTM) neural network.

Transfer learning strategies were adopted in two works by pre-training the CNNs used to classify the UGIE frames using images from the ImageNet dataset [48,49], thus providing enhanced robustness against overfitting. In addition, data augmentation techniques were applied by transforming the training frames using random cropping, rotations, and flips [26], translation, shears, and flips [48], as well as random jittering [49].

A simple preprocessing technique was adopted by [26] which consisted in cropping the surrounding black frames of the UGIE. As for the case of single-frame classifiers, some of the approaches consisted of a first CNN used to discriminate between frames collected outside or inside the patients [45,49].

Insights on the functioning of the deployed deep learning models have been inspected in two works by using CAM-based saliency methods for explainable AI [26,48].

It is possible to observe that the set of algorithmic solutions adopted to harness the information contained in the temporal dimension of UGIE videos for anatomical landmark detection is also quite heterogeneous and characterized by different attempts in combining CNN outputs with sequential models. The most promising results seem to be obtained when considering deep learning architectures specifically designed to cope with temporal sequences (see Table 2), as the LSTM model used in [49]. Efforts towards more interpretable and explainable modes are mainly limited to the adoption of CAMs for the CNN outputs, thus ignoring possible methods able to shed light on the inner operations of the models that also consider the temporal dimension. Finally, generalization issues are mitigated via the common adoption of data augmentation and transfer learning schemes.

##### Datasets

As it can be seen in Table 2, all the considered works used private data to train and validate the models. As for the case of single-frame anatomical landmark classification, the datasets used to train and validate multi-frame approaches showcased significant differences among them. Regarding the size of the datasets, those range from a minimum of 2599 labeled frames [26] to a maximum of 170,297 frames [49].

Moreover, different levels of complexity in the recognition of anatomical locations were considered, starting from approaches limited to the classification of a few landmarks (e.g., 6 landmarks in [48]), to compliance with the ESGE standard [26] and the Japanese systematic screening protocol (26 landmarks) [45], up to a maximum of 31 landmarks of a custom protocol [49]. A N/A class was also added to include background or non-informative frames in three works [45,48,49].

All four considered works were trained to detect and classify anatomical landmarks from WLI frames and occasional NBI images were relegated to the N/A class [48]. Although trained with WLI frames, the system described in [49] was shown to be able to classify anatomical landmarks collected with NBI during testing. Even though it is not explicit in their exclusion criteria, the work by Choi et al. [26] does not display results regarding non-WLI data.

As observed for the case of single-frame anatomical landmark detection, the datasets used to train and test the proposed models differ significantly in terms of the definition of classes and image modalities adopted. On the other hand, multi-frame approaches reflect more mature research efforts in anatomical landmark detection. These seem to converge to a more comprehensive and stable definition of the classification problems addressed, including an increasing number of landmarks that clearly reflect photodocumentation protocols.

##### Results

A comparative analysis of the raw performance results obtained by the four considered approaches is not possible, due to the diversity of the classification tasks considered. However, it is possible to observe a clear trend testifying that the exploitation of time correlation models in multi-frame classification approaches leads to better classification performance also in the presence of a higher number of anatomical landmark classes when compared to single-frame approaches. As an example, the IDEA system described in [49] achieves an average accuracy of 95% in classifying 31 different anatomical landmarks. In general, methods based on temporal deep learning systems outperform simple sequential classification models. These are affected by significant classification errors regarding the esophagus and the gastroesophageal junction [26]. In some cases, they were shown to base their classification output on non-relevant image features, for example when the weights of the CNN were almost exclusively associated with the presence of the endoscope itself in the retroflex view [48].

### 3.2. Lesion Detection in UGIE

**Figure 5:**
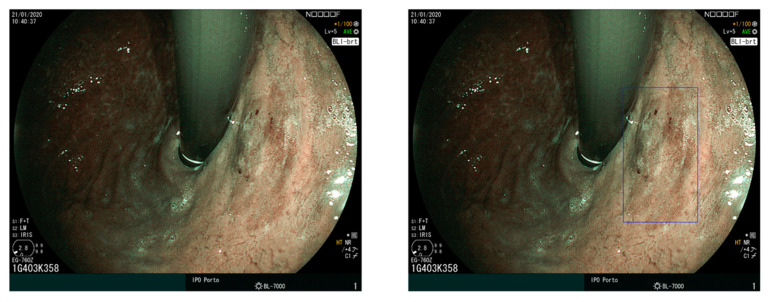
(**Left**) An early gastric cancer lesion missed by an endoscopist and (**Right**) correctly identified by the convolutional neural network by bounding box regression model by [52,53]. Note that, not only is the CNN’s prediction cued from subtle textural changes in the mucosa, but also that the output is a plausible bounding box around it. (Adapted from data kindly provided by Ishioka et al. [52]).

The detection of all existing lesions is the goal of UGIE. Since early lesions are often subtle and difficult to detect for human eyes, the exposure of all the mucosa may not suffice to guarantee that every lesion is detected, and AI can also have a role in improving lesion detection. An example is reported in Figure 5, which shows an early gastric cancer lesion missed by an endoscopist and correctly identified by the convolutional neural network algorithm presented in [51,52]. If we distinguish between lesion detection (*Are there any lesions in this UGIE exam? If so, where are these lesions?*) and lesion characterization (*How does each lesion contribute to a diagnostic decision?*), and accept that non-visualized lesions are part of the exam quality challenge and not the lesion detection challenge, what is the clinical motivation for this AI challenge? In fact, expert gastroenterologists typically exhibit very good performance in a lesion detection task if the lesion is correctly visualized.

However, by thinking further into the future, success in this challenge can definitely pave the way for enhancing the responsibility and support of physicians in training, and also create tools for more cost-effective screening strategies for GI cancer management. Besides, assistance in lesion detection may be important for community endoscopists outside expert centers [54]. Examples of these include the creation of the role of CAD-assisted gastroenterology technicians or, more ambitiously, fully automated screening using capsule endoscopy. Highly qualified endoscopists are short in supply [40] and are costly to train [26,54].

Given such a motivation, this section will revise current state-of-the-art approaches implemented in CAD systems that support lesion detection. A summary of the works that have proposed lesion detection approaches can be found in Table 3.

**Table 3:** Lesion detection algorithms.

Authors	Classes	Algorithm	Dataset	Image Modality	Results	AI Impact (Clinical Setting)
*Zhang* et al., *2020* [55]	Non atrophic gastritis Atrophic gastritis	DenseNet121	*(Private Dataset)* 3042 atrophic gastritis images 2428 normal images	WLI	Accuracy:94% Sensitivity: 95% Specificity: 94%	*N/A*
*Hirasawa* et al., *2018* [53]	EGC	SSD	(*Private Dataset*) 13,584 EGC images	WLI NBI Indigo carmine	Sensitivity: 92% PPV: 31%	*N/A*
*Yoon* et al., *2019* [56]	non-EGC EGC	VGG-16	*(Private Dataset)* 1705 EGC images 9834 non-EGC images	WLI	Specificity: 98% sensitivity: 91%	*N/A*
*Yan* et al., *2020* [57]	Intestinal Metaplasia (IM)	Xception NASNet EfficientNetB4	(*Private Dataset*) 1048 IM images 832 non-IM	NBI Magnifying NBI	EfficientNetB4: Sensitivity: 93% Specificity: 85% Accuracy: 88%	*N/A*
*Wu* et al., *2021* [58]	neoplastic lesions non neoplastic lesions	YOLO v3 and ResNet50	*(Private Dataset)* For YOLO v3: 15,341 lesion images 9363 non-lesion images For ResNet50: 4442 gastric neoplastic images 3859 non-neoplastic images	WLI	Accuracy: 89%; Sensitivity 92%; Specificity 88%	(Multi-center prospective controlled trial) Experts (n = 8): Sensitivity: 81% Specificity: 75% Accuracy: 78% Seniors (n = 19): Sensitivity: 84% Specificity: 77% Accuracy: 80% Juniors (n = 19): Sensitivity: 84% Specificity: 66% Accuracy: 73% ENDOANGEL: Sensitivity: 88% Specificity: 93% Accuracy 91%

#### 3.2.1. UGIE Lesion Detection in the Stomach

Most common pathological forms of gastric cancer are intestinal-type gastric cancers commonly associated with the presence of *Helicobacter pylori*-induced chronic inflammation, gastric atrophy (GA), and intestinal metaplasia (IM). These precancerous conditions correlate with a higher risk of cancer formation [59,60,61,62]. In this section, we will review state-of-the-art algorithms able to identify gastric lesions from UGIE videos.

##### Algorithms

The two main methodologies for the task of stomach lesion detection are: (i) *event detection*, classifying an UGIE video frame/image according to the presence of gastric lesion; (ii) *object detection*, extending these methods to also include information about the spatial location of the lesion in the image by means of a bounding box, in an object detection paradigm. Event detection algorithms are commonly based on the use of well-known deep convolutional neural networks such as the DenseNet [54], the VGG-16 [56], and the ResNet50 model. The authors of [57] trained Xception, NASNet, and EfficientNetB4 models to detect gastric IM achieving the best performance with the last model. Of particular relevance and in contrast with the other works considered, [56] proposed two different learning frameworks to train a VGG-16 CNN. In the first one, a standard cross-entropy loss between the predicted class and the ground truth label was adopted. A second learning approach defined a novel loss function that included a term accounting for the values of gradient-weighted class activation maps (Grad-CAMs), thus promoting the use of fine-grained features by the model when attempting to detect the presence of early gastric cancer (EGC) lesions.

No specific image preprocessing steps are explicitly described for the methods in [56,58]. In contrast, [55] used a specific algorithm to remove the patient information present in the UGIE videos, such as personal information and watermarks, that could be used by the CNN as a feature for lesion detection.

In order to increase the interpretability and explainability of model results, [56,57] used Grad-CAMs responsible for highlighting fine-grained features of the EGC lesions. The authors of [55] used class activation maps to indicate the features of the lesion that the CNN focused on.

Transfer learning techniques are largely used in both the event detection and object detection approaches. Namely, the CNN architectures used in all the three works mentioned are pre-trained using images from the ImageNet and COCO datasets, providing a good initialization of weights and enhanced robustness against overfitting.

Concerning object detection methodologies, lesion localization often adopts standard algorithms and architectures, such as the YOLO v3 [58] and the SSD [53] (see Figure 5) for detecting EGC. Both algorithms are one-stage detectors.

##### Datasets

All the considered works used private data to train and validate the models. The authors of [55] used a dataset of a total of 5470 gastric antral images from 1699 patients focusing only on atrophic gastritis images. Similarly, the dataset used by [56] included 11,686 images of healthy mucosa or containing lesions associated with EGC. On the other hand, the lesion detector developed in [58] used a dataset of more than 24,000 labeled images containing different kinds of lesions, including gastric neoplastic and non-neoplastic images. The authors of [53] collected more than 13,000 EGC images and [57] acquired more than 1000 IM images.

Image selection processes were adopted to generate the datasets used in [55,56]. In particular, [55] excluded the images affected by artifacts created by mucus, poor focus, insufficient contrast, motion-blurring, and gastric cancer. Similarly, images affected by motion-blurring, out of focus, halation, or poor air insufflation were excluded from the dataset used in [56]. The authors of [55,56,58] acquired only white-light endoscopy images. The authors of [57] only used NBI images (both magnified and non-magnified), and [53] selected WLI, NBI, and indigo-carmine images.

As previously observed in Section 3.2 and Section 3.3, pre-selected datasets can possibly link to sources of bias and limitations to the practical application of the developed methods of lesion detection.

##### Results

Heterogeneity in the kinds of gastric lesion classes considered in the different works considered makes it difficult to perform a fair comparison of their performance. The reduced intra-class variability associated with a dataset that considers only lesions of a single kind, for example, is bound to represent a simpler classification or object detection problem to be solved with a deep architecture.

Two of the three works presented only use classification models [55,56]. On the other hand, a recent work [58] considers both an object detection architecture and a classifier. An interesting strategy proposed in this work was that of training the classifier separately from the object detection module. This allowed the integration of the features extracted by the fine-tuned classifier in the object detection module. This approach seems especially promising as it shows highest detection performance (sensitivity of 96%) in the detection of gastric lesions within the surveyed body of work even when tested in an external dataset. The specificity, accuracy, and positive predictive value (PPV) achieved by this system were significantly higher (93%, 91%, and 90%) than those of the endoscopists (72%, 76%, and 71%), whereas the sensitivity and negative predictive value (NPV) were slightly higher compared with those of the endoscopists. Additionally, this system also reached an accuracy, sensitivity, and specificity of 89%, 90%, and 89%, respectively, when tested over an external dataset, proving the robustness of the model.

### 3.3. Lesion Characterization in UGIE

After successfully detecting that a lesion is present in a UGIE exam, we need to understand its relevance for the clinical decisions that will follow. This task is much more challenging than the previous one for a clinician since it depends on subtle visual changes that our human visual system is not fully prepared for. These include very subtle changes in color, texture, shape, and other visual structures that may only be identified using special lighting modalities such as NBI or by applying IC solutions to enhance the visual characteristics of the *villi* in the UGI walls. On a typical UGIE exam, the gastroenterologist is required to decide, in real-time, if the lesion is relevant and should be included in the report, and if he will extract a biopsy of the lesion or not. Deep learning support for this task can lead to at least two benefits: firstly, enabling the expert to have a second opinion in real-time if no other expert or telemedicine structure is available at that time. Secondly, creating a virtual biopsy that is instantaneous, thus enabling dense sampling of the tissue and reducing the number of unnecessary invasive interventions.

The same structure from the previous sections is followed: we will revise current state-of-the-art approaches implemented in CAD systems that support lesion characterization in UGIE exams. A summary of the works that have proposed lesion characterization approaches can be found in Table 4.

**Table 4:** Lesion characterization algorithms.

Authors	Classes	Algorithm	Dataset	Image Modality	Results	AI Impact (Clinical Setting)
*Wu* et al., *2021* [58]	Invasion depth: mucosal and submucosal Differentiation Status: Differentiated and undifferentiated	ResNet-50	*(Private Dataset)* * 3407 images of gastric cancer 1131 differentiated type images 1086 undifferentiated type images	WLI Magnifying NBI	EGC invasion WLI: Accuracy: 88%; Sensitivity: 91%; Specificity: 85% EGC differentiation M-NBI: Accuracy of 86%; Sensitivity: 79%; Specificity: 89%	(Multi-center prospective controlled trial) EGC invasion Experts(n = 8): Sensitivity: 57%% Specificity: 76% Accuracy: 69% EGC invasion Seniors (n = 19): Sensitivity: 60% Specificity: 66% Accuracy: 64% EGC invasion Juniors (n = 19): Sensitivity: 61% Specificity: 61% Accuracy: 61% EGC invasion AI predictions: Sensitivity: 70% Specificity: 83% Accuracy 79% EGC differentiation Experts(n = 8): Sensitivity: 47% Specificity: 83% Accuracy 72% EGC differentiation Seniors (n = 19): Sensitivity: 53% Specificity: 74% Accuracy: 67% EGC differentiation Juniors (n = 19): Sensitivity: 56% Specificity: 60% Accuracy: 59% EGC differentiation AI predictions: Sensitivity: 50% Specificity: 80% Accuracy 71%
*Yoon* et al., *2019* [56]	T1a T1b Non-EGC	VGG-16	*(Private Dataset)* 1097 T1a-EGC 1005 T1b-EGC 9834 non-EGC	WLI	Specificity: 75% Sensitivity: 82%	*N/A*
*Nagao* et al., *2020* [63]	M-SM1 SM2 or deeper	ResNet-50	*(Private Dataset)* 10,589 M-SM1 images 6968 SM2 or deeper images	WLI NBI Indigo	WLI Accuracy: 95% NBI Accuracy: 94% Indigo Accuracy: 96%	*N/A*
*Zhu* et al., *2019* [64]	P0 (M or SM1) P1 (deeper than SM1)	ResNet-50	*(Private Dataset)* 545 P0 images 245 P1 images	WLI	Accuracy: 89% Sensitivity: 76% Specificity: 96%	CNN Accuracy: 89% Sensitivity:76% Specificity: 96% Experts(n = 8): Accuracy: 77% Sensitivity: 91% Specificity: 71% Junior (n = 9): Accuracy: 66% Sensitivity: 85% Specificity: 57%
*Xu* et al., *2021* [65]	GA IM	VGG-16	*(Private Dataset)* 2149 GA images 3049 IM images	Magnifying NBI Magnifying BLI	GA Accuracy: 90% Sensitivity: 90% Specificity: 91% IM Accuracy: 91% Sensitivity: 89% Specificity: 93%	(Multi-center Prospective blinded trial) GA classification CAD System: Accuracy: 87% Sensitivity: 87% Specificity: 86% Experts(n = 4): Accuracy: 85% Sensitivity: 91% Specificity: 72% Non experts (n = 5): Accuracy: 75% Sensitivity: 83% Specificity: 59% IM classification: CAD System: Accuracy: 89% Sensitivity: 90% Specificity: 86% Experts(n = 4): Accuracy: 82% Sensitivity: 83% Specificity: 81% Non experts (n = 5): Accuracy: 74% Sensitivity: 74% Specificity: 73%

* An external dataset of 1526 images has been used to test the performance of the model.

#### 3.3.1. UGIE Lesion Characterization in the Stomach

Gastric lesion characterization using deep learning techniques is extremely important in order to provide vital information to the endoscopist regarding the necessity of further actions to be performed during the examination (e.g., endoscopic resection, need for endoscopic surveillance) and to assist in diagnosis. This section focuses on algorithms that have been developed to provide information regarding gastric lesions, in terms of their invasion depth and differentiation status.

##### Algorithms

**Figure 6:**
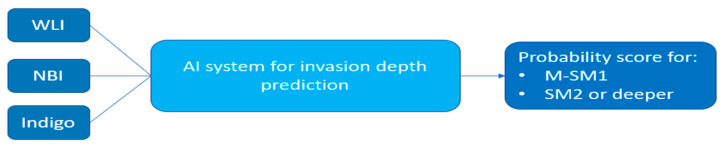
Schematic illustration of the method proposed in [63] that uses a ResNet50 model to classify the gastric lesion invasion depth in WLI, NBI, and indigo carmine images (adapted from [63]).

The tasks associated with gastric lesion characterization in the three works considered in this section involve the assessment of the invasion depth and the differentiation status. In particular, [58] developed two approaches for different types of EGC characterization, namely, predicting EGC invasion depth in WLI and predicting EGC differentiation status in magnifying NBI. Two works proposed a method for predicting gastric cancer invasion depth [63,64] (see Figure 6). In addition, [56] applied the same methods described in Section 3 for lesion detection to predict the invasion depth of EGC as well.

Additionally, one study predicted precancerous gastric conditions by classifying lesions as GA and IM in magnifying NBI and BLI [65].

All these works made use of the well-known ResNet models to implement image classification, with weights obtained by pre-training over the ImageNet dataset [66]. Exception to this were [56,65] which used a pre-trained VGG-16 model.

Regarding preprocessing, [64] resized all the available images for training to a dimension of 224 × 224 pixels to fit the original input size designed for the ResNet model. After this, training images were augmented using random vertical and horizontal flips and scaling. Standard preprocessing steps were also adopted by [64,66], which applied mean subtraction and normalization to each image before passing it to the network. Then, simple data augmentation was applied via random image rotations and flips.

Regarding the interpretability of the proposed classification models, [64] performed an occlusion analysis to find which area of the images was most important to the classification output. Occlusion regions of dimension 60 × 60 were slid from top-left to bottom-right with a stride of six pixels to generate a new dataset with different patches occluded. Heatmaps were produced showing the output classification probabilities of the different regions of the image.

Commonly adopted algorithmic approaches for gastric lesion characterization rely on the use of standard ResNet models that do not represent the current state-of-the-art in several computer vision tasks. Multi-task and joint end-to-end learning frameworks are not considered, thus revealing a potential margin for the exploration of more sophisticated deep learning approaches in this area. On the other hand, the occlusion analysis performed by [64] provides an interesting contribution towards model interpretability, without relying only on CAM-based explainable AI solutions, that can deal with functions that are flat, without or only with very small gradients.

##### Datasets

All the datasets in the considered works are private. Dataset sizes vary from a few hundred (790 in [64]) to several thousand (16,558 in [63]).

Regarding invasion depth estimation, both [63,64] define two classes based on grouping over standard clinical characterizations of gastric lesions. They start from a characterization of invasion depths defined over five classes: mucosa (M), submucosa (SM), *Muscularis propria*, subserosa, and serosa. The SM can be further subclassified as SM1 (invasion depth of <0.5 mm of the *Muscularis mucosae*) or SM2 (invasion depth of ≥0.5 mm of the *Muscularis mucosae*). Then, the two classes considered by the algorithms were defined as P0, corresponding to an invasion depth restricted to M or SM1, and P1, corresponding to an invasion depth deeper than SM1. A binary characterization of invasion depth is considered in [58], where one class is associated with lesions whose invasion is limited to the mucosa and another class is attributed to lesions whose invasion reaches the submucosa.

For the GA and IM classification, [65] collected an internal dataset for training the model (2149 GA images and 3049 IM images) from two different hospitals, plus one external test set to evaluate the model (344 GA images and 708 IM images) from three different hospitals. In addition, a prospective video test set was acquired (37 GA videos and 61 IM videos) and the AI-based system performance was compared to that of the endoscopists.

Concerning frame selection, images affected by poor observation conditions, such as blurring, food retention, bleeding, and insufficient air insufflation were discarded in all considered articles. In addition, ref. [64] excluded images from patients who underwent gastrectomy, lesions that were considered difficult to see in magnifying NBI, cases of pathologically confirmed gastric cancer, and abnormalities in the submucosa. Moreover, ref. [63] also excluded images with gastric adenocarcinoma of fundic gland type, images from patients with a historic treatment for the target lesion, and multiple lesions that were seen simultaneously in the same endoscopic picture. The authors of [65] excluded images without pathological diagnosis, esophageal lesions, duodenal lesions, cancers, high grade dysplasia, low grade dysplasia, polyps, and blurred or not clear images.

All the considered works used WLI data. In addition, ref. [58] also included magnifying NBI images in the analysis of the differentiation status, and [63] used non-magnifying NBI images and indigo carmine dye contrast imaging. The authors of [64] only used magnifying imaging, namely magnifying NBI and BLI.

##### Results

The works describing the characterization of gastric lesions employ deep learning methods designed for classification purposes. ResNet architectures are common throughout all the surveyed articles. This architecture implements skip connections that allow for the gradient to be more efficiently backpropagated, which is of special importance when the number of hidden layers is high. The success of [58,63,64] serves as an indicator of the reliability of the ResNet design. The ResNet-50 model achieved accuracy levels above 85% when classifying invasion depth in the mucosa and submucosa layers (M-SM1 and SM2). An analysis is made by [63] using the separate invasion depth classifications results on WLI, NBI, and indigo carmine modalities. Different features are highlighted within each modality experiment, with indigo carmine reaching the highest accuracy of 96%, followed by the WLI with 95% and NBI with 94%.

The authors of [64] conducted a thorough analysis in the validation of their model for the tasks of GA and IM classification. They used 3 independent sets: the first one, collected from two institutions, was split into a train, validation and (internal) test sets, and was used to develop the model. The second one, collected from 3 institutions, served as an external test set. Finally, a prospective single-center external test dataset containing 71 precancerous and 36 control images served as a benchmarking for the comparison of the algorithm with trained endoscopists. Concerning the GA task, the system measured an accuracy of 90%, sensitivity of 90%, and specificity of 91% in the internal test set. In the external test set, the DNN measured 86% accuracy, 90% sensitivity, and 79% specificity. Compared to the measurements of the internal test set, there was a significant decrease in specificity (11%), thus serving as evidence that the model is prone to output false positives. This was also reflected in a variation of accuracy of approximately 6%. Regarding IM, 91% accuracy, 89% sensitivity, and 93% specificity were observed in the internal test set. When evaluated in the external test set, the measurements were 86% accuracy, 97% sensitivity, and 72% specificity. Contrary to the proportions observed on the internal test set, the system shows disparate measures from the internal test set, outputting predictions that are more sensitive but less specific. This apparent contradiction in the experiment can potentially be caused by data drift from the internal to the external dataset, which in turn can be explained by data collection or labeling mistakes. Another hypothesis is a lack of representative data in the internal dataset, thus resulting in overfitting to the internal test set. Finally, in the benchmarking external test set, 5 non-expert and 4 expert endoscopists enrolled in the tasks of classifying GA and IM. For both tasks the model achieved a performance similar to clinical experts. Concerning the GA task, the model outperformed non-expert clinicians, measuring 75% accuracy, 59%, specificity, and 80% PPV. The same trend was observed for the IM classification task, where the system outperformed non experts in all the measured metrics, displaying 74% accuracy, 74% specificity, 85% PPV, and 59% NPV. Again, although the results when benchmarking with humans are favorable to the algorithm, there is a clear discrepancy in performance in the different datasets for each experiment. Since the algorithm showed decent performance in the internal test set, the most probable explanation for this mismatch in performance is likely due to bias in the internal dataset used to develop the model.

## 4. Discussion

Based on the findings reported in Section 3, in this section, we describe the main trends revealed by the analysis of the works reviewed for exam quality assessment, lesion detection, and lesion characterization. Moreover, for each of these areas, we set the pillars for a roadmap towards the application of AI-assisted UGIE in clinical practice.

### 4.1. UGIE Exam Quality Assessment

Artificial intelligence methods for automatic UGIE exam quality assessment have mainly been focused on assessing the completeness of the exam by leveraging the definitions of different photodocumentation protocols. They consisted mainly in the implementation of algorithms able to automatically detect standardized anatomical landmarks and provide real-time information about the regions of the upper GI tract that have been visited during the examination.

The methods applied for these tasks have seen a rapid evolution in their performance when shifting from a single-frame to a multi-frame paradigm with the ability to leverage information contained in correlated frame sequences. Most of the approaches considered deep learning solutions based on well-known CNN architectures and recurrent neural networks for time sequence modeling. Multi-frame classification methods have demonstrated high accuracy in recognizing larger numbers of anatomical landmarks (e.g., 26 in [39], 31 in [42]). The machine learning solutions adopted by the current state-of-the-art mainly used deep learning techniques that have been adopted in several computer vision and machine learning problems for almost a decade now. In addition, further efforts could be made to improve model generalization and explainability, which are addressed only partially in the UGIE exam quality assessment literature, as observed in Section 3.1.1 and Section 3.1.2. Moreover, the datasets used to train and validate the models are all private and significantly heterogeneous in terms of both definitions of anatomical landmarks and imaging modalities.

In this sense, there is a clear opportunity for the application of more modern and sophisticated architectures that could be able to provide increased robustness against non-idealities in the video collection as well as different image quality levels. Novel contributions in the development of machine learning algorithms for anatomical landmark detections can be envisioned in the following four directions:

1. Beyond CNNs: The adoption of novel deep learning architectures for visual understanding, as the recently proposed visual transformers [67], could provide a significant improvement at the backbone of the multi-frame classification approaches. This is due to their improved capacity in automatically selecting relevant features from the imaging data as well as better capacity in transferring efficiently information from a larger receptive field into deep layers.
2. Hybrid models: The sequential nature of UGIE examinations and, in particular, the strong priors associated with the expected order in visiting the different anatomical landmarks open up the possibility of pairing highly discriminative deep learning models with robust time sequence statistical models able to model more explicitly time evolution [68]. These techniques are then expected to provide models that are more robust to input perturbation than purely data-driven methods, while at the same time requiring a lower amount of training data to generalize well.
3. Enhanced explainability: Efforts to provide explainable and interpretable models for anatomical landmarks detection have focused mainly on the use of CAM-based approaches able to highlight which part of the considered frame is mostly responsible for the output of the CNN classifier. Increased explainability should be pursued, by starting with the inclusion of the temporal dimension in substantiating why a given landmark is detected by the algorithm at that particular time of the UGIE procedure. The produced explanations could be also enriched with example-based approaches able to showcase, in real-time, to the operator to most similar and significant landmarks encountered in other exams [69].
4. 3D Photodocumentation: Assuming screening protocols are followed, machine learning algorithms can project the frames of an UGIE video onto a three-dimensional reconstruction of the UGI tract. These algorithms can potentially be applied to any photodocumentation protocol since they can in principle construct a complete representation of the UGI tract. They also allow one to query a bounded region of the produced 3D surface and retrieve the original 2-D frames used in the projective step. Not only is this functionality relevant from a usability perspective, but it can also convey a more accurate realization of the mental model required by endoscopists when interpreting a UGIE report. Blind spots become overwhelmingly evident in a 3D model, potentiating feedback loops for the physicians. The work of Widya et al. [70,71], which is based on classical methods for structure invariant feature transforms (SIFT) and structure-from-motion, has been seminal in this direction, leading towards reconstructing a virtual 3D model of the stomach from unlabeled UGIE data. More recently, Yuwei Xu et al. [72] introduced a CNN that is able to find more invariant anchor points that enable 3D reconstructions of higher resolution and under more challenging conditions. Moreover, advancements in the field of light field imaging and neural radiance fields are likely able to mitigate incomplete reconstructions through 3D view synthesis [73,74,75,76].

Regarding datasets and the definition of homogeneous anatomical landmark detection tasks, the availability of large, public, annotated datasets with information labels regarding anatomical landmarks in UGIE videos would potentially boost and foster research efforts in this area. In addition, defining anatomical landmark classification methods that are more adherent to the official photodocumentation protocols put forward for UGIE quality control, combined with public datasets, would allow fair and transparent comparisons of different approaches. This would provide the means for the rapid evolution of the current approaches towards increased robustness and reliable applicability in clinical practice.

### 4.2. Lesion Detection in UGIE

Automatic lesion detection in the gastric tract has attracted considerable interest from the research community, which has produced several efforts in adapting and applying state-of-the-art deep learning models for image classification and object detection to the analysis of UGIE videos. The results reported by these approaches are usually characterized by high accuracy as shown in recent meta-analysis works [5,6]. However, most of the works are tested only considering UGIE videos containing a small number of different lesions, which can point to a possible gap to be bridged before the practical adoption of such algorithms. In addition, using data from institutions/hospitals and validating the model in the same type of images can create availability bias. To overcome this problem and train more general models, the data acquisition should be focused on more representative data and a more heterogeneous collection of samples. There is also a lack of studies that include endoscopists in the AI experiment loop. Experiments measuring if a human supported by AI is less likely to miss lesions are an important first step in this direction. Furthermore, direct comparison of the AI systems alone, especially versus highly experienced clinicians, can benefit both the development of more sophisticated algorithmic solutions and potentially highlight systematic mistakes that can still affect specialists in the field. Regarding the algorithmic design, based on the state-of-the-art works collected, suitable next steps would be focused on more explainable models that can explicitly highlight features and attributes correlated to the model decision. In this sense, robust algorithmic approaches need to be further explored. In particular, the following aspects can be envisioned as possible pillars of the algorithmic innovation in lesion detection systems:

1. Beyond CNNs: Similar to what has been discussed for anatomical landmark detection, novel architectures for computer vision tasks can be considered in the design of lesion detection pipelines, also including visual transformers. As an example, [77] uses self-attention mechanisms of transformers which enables the models to selectively focus on certain parts of their input and thus reason more effectively. This type of architecture that relies on attention mechanisms also helps in improving the interpretability of the model.
2. Generative models for data augmentation: To overcome the lack of publicly available data and the representativeness of multiple lesions classes, generative models can synthesize images of lesions. Generative models can also be used for explaining the attributes that are highly correlated with the model’s decision; for example, [77] trained and highlighted the most relevant attributes for the predictions. StyleGAN [78] models can learn the classifier-specific style space as explanatory attributes.
3. Unsupervised and semi-supervised approaches: The performance of the deep learning solutions heavily depends on the quality of the data used for training. Possible new directions to guarantee better generalization of lesion detection models could lie in the usage of unsupervised and semi-supervised learning techniques that could leverage the huge amount of non-labeled UGIE video currently available. These can be achieved by considering first unsupervised feature extraction approaches based on the use of autoencoders or generative adversarial networks.
4. Use spatial information: A possible approach to provide more reliable detection performance is envisioned by embedding prior spatial information into the lesion detection algorithm. For example, CAD systems that provide automatic photodocumentation assistance via anatomical landmark detection could use such detailed information regarding the UGI navigation process, thus providing context information to the lesion detection algorithms.

A fundamental challenge to overcome in this area is data availability. Public, large, representative datasets will allow the quality of the different lesion detection approaches proposed in the literature to be properly assessed.

### 4.3. Lesion Characterization in UGIE

Automatic gastric lesion characterization is particularly important since it enables to assess invasion depth, dimension, and differentiation status before performing endoscopic resection. The main research regarding this problem leverages deep learning techniques for image classification and region segmentation. Reported results are promising, but some limitations can still be observed. As for the case of lesion detection, the data collection can be biased since most datasets are collected from single centers. Moreover, the images are sampled from a reduced number of patients/cases. Explainability efforts should continue to be made to validate the prediction from the black box models dominant in the literature.

There is also room for improvement concerning the algorithmic approaches. Below we list a set of possible innovations for lesion characterization systems:

1. Explainable CNNs: Model explainability is one of the main focal points for future research, due to the clear necessity of interpretable and transparent models when applied to sensitive applications domains, such as healthcare. Visual transformers can naturally enable model interpretability by leveraging self-attention layers.
2. Generative models: The lack of available data and the representation of each class of interest is also a common problem pointed out in several sections of this review. Generative models can overcome this issue by creating synthetic images that enhance the representativeness of classes within the dataset. Beyond that, styles can be transferred from, for example, different image modalities, thus creating synthetic NBI images from WLI or synthetic indigo carmine dye contrast images from WLI, and so on. To this end, conditional generative adversarial approaches, such as the StyleGAN [78] can be used.
3. Beyond 2D Characterization: Segmentation models can be used to precisely characterize the extension of the region of mucosa affected by a lesion. A possible evolution to this approach would be that of providing a description of the 3D volumetric characteristics of the abnormal tissue, which can give more information about the invasion depth throughout the lesion volume, thus providing a further step towards the actual implementation of what could be interpreted as a sort of 3D visual biopsy.

## 5. Conclusions

AI support for UGIE exams represents a rapidly evolving area that has been able to attract the attention of the clinical community in gastroenterology with unprecedented results. Research approaches surveyed in this work have mainly focused on three fundamental areas of UGIE examination: exam completeness, lesion detection, and lesion characterization. Regarding quality control, automatic anatomical landmark detection systems able to harness the information contained in the temporal dimension of UGIE videos represent currently the most successful approaches. Several systems have also focused on lesion detection, thus providing information about which frames of the UGIE video contain lesions and about their spatial localization, by leveraging different deep learning architectures for object detection. Promising approaches able to combine both spatial and temporal features have been presented for this task. Current examples of lesion characterization systems also provide information regarding the extension of such lesions (via image segmentation), their invasion depth, and status.

Although the results reported in the literature often show a high potential of these approaches, algorithmic limitations common to the approaches in all three mentioned areas can still be observed. These include the adoption of mainly standard CNN architectures without significant design adaptations to the specific tasks at hand and reduced efforts in providing interpretable models, which are mainly limited to saliency methods, such as CAMs. In addition, from a data point of view, the constant adoption of only private datasets and their selection criteria point to potential sources of different kinds of bias that can invalidate in part the validity of the high accuracy values reported. This fact can possibly hinder a fully reliable adoption of CAD-assisted endoscopy in clinical practice.

Future research directions have been identified in the roadmap towards more reliable and robust CAD UGIE algorithms. These include the exploration of more recent deep learning architectures, e.g., transformer models and hybrid model-based/data-driven approaches, as well as the use of unsupervised and semi-supervised learning frameworks, etc. Methods able to combine efficiently domain knowledge (possibly coming from anatomical landmark detectors) in lesion detection/characterization tasks are expected to better mimic the ability of expert endoscopists in coping also with highly non-ideal imaging conditions. In addition, the availability of large publicly available UGIE datasets, with annotations aligned to standard protocols, is expected to foster new research efforts and boost further the evolution of the field. Finally, more disruptive future lines of research have been suggested, such as one involving the evolution of current methods for dynamic 3D reconstruction of the gastric internal surface from UGIE video, thus presenting a potential paradigm shift in anatomical landmark detection and UGIE exam control.

## Data Availability

Not applicable.
